# Efficacy in Clinical Studies of a Skin Care Serum Formula Containing 7 Short and Long Saccharides and Bacterial Fractions to Repair and Strengthen the Skin Barrier From Aggressions in Intense Conditions

**DOI:** 10.1111/jocd.70193

**Published:** 2025-05-03

**Authors:** Caroline Pichon, Marion Mesrobian, Sybille De Bussy, Elodie Valverde, Audrey Gueniche

**Affiliations:** ^1^ L'oréal Research & Innovation Chevilly‐Larue France

**Keywords:** aggression, bacterial extracts, prebiotic, serum, skin barrier, skin care formula, stable skin

## Abstract

**Background:**

A healthy skin barrier is essential to protect against the entry of harmful substances or the loss of internal fluids, especially water and electrolytes. Various aggressions are known to weaken the skin barrier.

**Aims:**

To evaluate single or multiple applications of a serum formula designed to mitigate skin barrier dysfunction and improve recovery after exposure to three different external aggressions (including one cumulative aggression under intense conditions).

**Patients and Methods:**

Five single‐blind, controlled, intra‐individual clinical studies in healthy subjects evaluated the effect of serum formula vs. untreated skin after aggression with harsh cleanser, emery paper abrasion, or tape stripping followed by exposure to 4°C or 40°C. Skin barrier function was assessed by transepidermal water loss (TEWL) and skin pH.

**Results:**

After harsh cleanser aggression, significant improvements in pH recovery and TEWL were observed after serum application, vs. untreated control, indicating significantly faster skin barrier recovery. Similarly, after tape stripping followed by intense cold or hot conditions, barrier function recovery was also significantly faster with an application of serum versus untreated control. In addition, serum‐treated skin was more resistant against abrasion after serum treatment compared to the untreated control.

**Conclusions:**

The serum formula, containing a unique combination of seven short and long saccharides and bacterial fractions, helped repair and strengthen the skin barrier to protect against abrasion. Taken together, these studies showed improved skin barrier recovery after exposure to different types of aggression (harsh cleanser or cumulative aggression by tape stripping followed by intense cold or hot conditions).

## 1 Introduction

The skin provides a physical and immunological barrier to the external environment. A healthy skin barrier is thus essential to protect against the entry of harmful substances, as well as the loss of internal fluids, especially water and electrolytes. The stratum corneum, tight junctions, and desquamation play a key role in the physical skin barrier, while the biochemical barrier is important for the regulation of an acid pH and appropriate levels of ceramides organized in lamellae [1] to limit transepidermal water loss (TEWL) [2]. Instrumental TEWL measurement can be used in vitro and in vivo to assess skin barrier function [3]. The skin innate immunity, including physical, molecular, and microbiome barriers, protects against invading pathogenic microorganisms, inflammation, oxidation, and helps restore and promote skin barrier recovery [4].

Internal (e.g., psychological stressors, sleep deprivation, poor nutrition) and external aggressors (e.g., air pollution, intense temperature and sunlight), whether acute or chronic exposure, are known to weaken certain skin functions and damage the skin barrier [5, 6]. Protecting the skin microbiota and providing a source of nutrients should therefore help strengthen the skin and promote faster recovery after aggression. Indeed, various prebiotic and bacterial fractions constituents in cosmetics have been shown to restore skin barrier function, prevent water loss, and improve skin stability [7].

The investigational serum formula evaluated in our studies contains seven short and long saccharides and bacterial fractions of *Bifidobacterium longum, Saccharomyces cerevisiae, Lactobacillus casei*, and *acidophilus* extracts, long polysaccharides and short sugars, hyaluronic acid, mannose, complex sugars of a biotechnologically derived alpha‐glucosaccharide, and a beta‐fructosaccharide extracted from yacoon root. The purification and fragmentation of biotechnologically derived bacterial fractions from living microorganisms provide an effective source of nutrients that are easily assimilated by the skin but also may stimulate the skin cells. In particular, *Bifidobacterium longum* fractions have been shown to stimulate markers of skin differentiation in vitro [8]. Furthermore, in a randomized double‐blind controlled clinical trial, *Bifidobacterium longum* fractions demonstrated a strengthening of the epidermal barrier and a decreased sensitivity [9]. Mannose has previously been shown in vitro to stimulate cell proliferation, increase the expression of Claudine 1, Transglutaminase K, and keratin 5, Corneodesmosin, Desmoglein 1, Desmocollin 1, and increase epidermal thickness [unpublished data]. Also, alpha‐glucosaccharide, beta‐fructosaccharide extracted from yacoon root in addition to *Lactobacillus casei* and *acidophilus* extracts induce, in vitro, an increase of several barrier function markers expression [unpublished data]. Additionally, *Saccharomyces Cerevisiae extract* (yeast extract) demonstrated an effect on the dermis by increasing the production of Collagen I, III, and Perlecan, in vitro [unpublished data].

The aim of the studies was to evaluate the efficacy of a unique serum formula containing short and long saccharides, bacterial fractions, and yeast extract designed to protect and repair the skin barrier after exposure to various external aggressions (harsh cleanser, mechanical abrasion and tape‐stripping followed by intense cold or hot environmental conditions).

## 2 Materials and Methods

### 2.1 Treatment Formula

In five clinical studies (see Table 1), all subjects received the same dermocosmetic serum formula containing seven short and long saccharides and bacterial fractions.

**TABLE 1:** Summary of the study design of five clinical studies performed to evaluate the impact of the serum formula on skin barrier function after various aggressions (harsh cleanser, mechanical abrasion and cumulative aggression by tape stripping followed by intense cold or hot conditions).

Aggression	Study location and dates	Number of subjects per protocol	Study design and assessments	Inclusion criteria	Site and skin type	Standardized skin care regimen	Exposure to aggression	Treatment with serum
Chemical aggression by harsh cleanser (Study 1)	France (09/2018–02/2019)	31 healthy women	Single‐blind controlled intra‐individual study. Instrumental assessments: TEWL and pH	European 55–65 years old Fitzpatrick skin type (phototype) II and III Post‐menopausal for at least 3 years Crow's‐feet wrinkles between 3 and 4 (Atlas score) Photoaging score between 0 and 6 (Griffith score) Skin tightness after harsh cleanser on whole face score ≥ 3 on a scale from 0 to 4 Body mass index 18.5—2S7 Non‐smoker or ≤ 3 cigarettes/day	Face All skin types	D‐28 to D0: wash‐out with standardized skin care (foaming gel, sunscreen, and neutral cream) D0 to D56: standardized skin care plus test serum	On D0, D14 and D56: Harsh cleanser applied for 1 min before rinsing and blotting dry. D‐3: Harsh cleanser applied for before‐treatment negative control	D0 to D56: serum application twice a day (plus standardized skin care regimen) vs. before‐treatment negative control
Chemical aggression by harsh cleanser (Study 2)	Singapore (09/2018–01/2019)	40 healthy women	Single‐blind controlled intra individual study Instrumental assessments: TEWL and pH	Chinese 55–65 years old Fitzpatrick skin type (phototype) II and III Post‐menopausal women Fine‐line wrinkles self‐assessed visibility on whole face (score of 4 on a scale from 0 to 9) Skin tightness after harsh cleanser on whole face self‐assessed score ≥ 3 (on a scale from 0 to 4) Lack of skin luminosity/radiance on whole face score ≥ 4 (on a scale from 0 to 9) Skin tone evenness on whole face score ≥ 4 on a scale from 0 to 9	Face All skin types	D‐28 to D0: wash‐out with standardized skin care (foaming gel, sunscreen, and neutral cream) D0–D56: standardized skin care plus test serum	On D0, D14 and D56: harsh cleanser applied for 1 min before rinsing and blotting dry D‐3: harsh cleanser applied for before‐treatment negative control	D0 to D56: serum application twice a day (plus standardized skin care regimen) vs. before‐treatment negative control

Aggression	Study location and dates	Number of subjects per protocol	Study design and assessments	Inclusion criteria	Site and skin type	Standardized skin care regimen	Exposure to aggression	Treatment with serum
Mechanical aggression by emery paper abrasion (Study 3)	France (12/2021–01/2022)	29 healthy women	Single‐blind monocenter, randomized, study Instrumental assessment: TEWL	European 18–60 years old Fitzpatrick skin type (phototype) II and III Hydration (corneometry) of < 40 a.u. TEWL ≥ 6 g/m^2^/h Delta of TEWL > 3 and < 12 after abrasion at D0 Not experiencing hot flushes	Forearm Self‐declared dry skin	Neutral cleanser throughout the study D‐7 to 0: wash‐out period	On D0 and D28: abrasion by applying constant pressure and 8 back‐and‐forth movements of emery paper (grain size 120)	D0 to D28: serum application twice a day on one forearm vs. untreated forearm control.
Aggression by tape stripping followed by intense cold and windy conditions (Study 4)	France (10/2022)	24 healthy women	Single‐blind non‐invasive, monocenter, randomized, instrumental assessment: TEWL	European 18–65 years old Fitzpatrick skin type (phototype) II and III TEWL ≥ 6 g/m^2^/h before stripping on the forearms TEWL ≥ 20 g/m^2^/h after stripping on the forearms Not experiencing hot flushes	Forearm Dry skin but not sensitive skin		Before serum application: Mean of 18–19 tape strips (D‐squam) per zone After serum application: 2 h exposure in intense cold and windy conditions (controlled room at + 4°C ± 1°C, 0.88 m/s wind, low hygrometry)	After tape stripping and before intense cold and windy conditions, single controlled application (2 mg/cm^**2**^) of serum on one forearm vs. untreated forearm control
Aggression by tape stripping followed by intense hot, dry and windy conditions (Study 5)	France (10/2022)	24 healthy women	Single‐blind non‐invasive, monocenter, randomized, Instrumental assessment: TEWL	European 18–65 years old Fitzpatrick skin type (phototype) II and III TEWL ≥ 6 g/m^2^/h before stripping on the forearms TEWL ≥ 20 g/m^2^/h after stripping on the forearms Not experiencing hot flushes	Forearm Dry skin but not sensitive skin		Before serum application: Mean of 18 tape strips (D‐squam) After serum application: 2 h exposure in intense hot, dry and windy conditions (controlled room at 40°C, 15% hygrometry)	After tape stripping and before intense hot and windy conditions, single controlled application (2 mg/cm^**2**^) of serum on one forearm vs. untreated forearm control

The serum composition includes mannose, *Bifidobacterium longum* lysate, polymnia sonchifolia root juice, *Saccharomyces cerevisiae* extract, alpha‐glucan oligosaccharide, *Lactobacillus* extracts, ascorbyl glucoside, salicyloyl phytosphingosine, tocopherol, sodium hyaluronate, adenosine, alcohol denat, PEG‐60 hydrogenated castor oil, glycerin, hydroxyethylpiperazine ethane sulfonic acid, ammonium polyacryloyldimethyl taurate, water, xanthan gum, pentylene glycol, dimethicone, disodium edta, octyldodecanol, preservative, and fragrance.

### 2.2 Study Assessments

Instrumental analysis performed by an expert evaluator on facial or forearm skin included hydration (only for inclusion criteria) using a Corneometer (Courage + Khazaka, Germany), TEWL using a Tewameter (Courage + Khazaka, Germany) and pH measurement using a skin pH meter Ph900 (Courage + Khazaka, Germany); all assessments were performed after prior stabilization for 30 min under controlled conditions (temperature 20°C to 23°C, hygrometry 50% ± 10%) before and after single or multiple applications of the serum formula.

### 2.3 Clinical Studies

Table 1 summarizes the study design of five clinical studies performed to evaluate the impact of the test serum formula.

In all cases described, we used a product in a neutral bottle; the volunteers were not aware of the type of product used, nor the technicians performing the measurements. Only the principal investigator and the sponsor were notified, but they do not interact directly with the measurement process or the volunteers.

Our blinding method is *single‐blind* with additional precautions to limit bias from those aware of the treatment group. The added measures of using blinded bottles and keeping the informed parties separate from the measurement process greatly strengthen the study design and reduce potential bias, even if it is not strictly double‐blind.

#### 2.3.1 Chemical Aggression by Harsh Cleanser (Studies 1 and 2)

Single‐blind, controlled, intra‐individual clinical studies were conducted in France (Study 1; Table 1) and Singapore (Study 2; Table 1) to evaluate the impact of the serum formula to protect against a harsh skin cleanser, as measured by instrumental evaluation of TEWL and skin pH on facial skin.

Thirty‐one European women who met inclusion criteria were recruited, aged between 55 and 65 years old (Study 1; Table 1). Forty Chinese women who met inclusion criteria were recruited, aged between 55 and 65 years old (Study 2; Table 1).

For 28 days before study start (washout period) and throughout the serum treatment period (D0–D56), subjects applied to their face a routine skin care regimen of the provided mild cleanser (marketed foaming gel, morning and evening), sunscreen (marketed SPF cream, morning), and neutral cream formulation (evening). In addition to the routine skin care, two fingertip units of the investigational serum formula were applied twice daily (morning and evening) from D0 to D56.

Subjects applied a specific marketed skin cleanser (1‐min wash before rinsing with tepid water and blotting dry with a sterile cloth) at the investigational center at five time points: on D‐28 to test if the subject was sensitive to the cleanser; on D‐3 as the before‐treatment negative control; and on D0, D14, and D56 (immediately after the harsh cleanser aggression) during the serum formula treatment period.

TEWL was assessed on the designated zone on the right cheek and pH was assessed on the left cheek before application of the harsh cleanser and then immediately after, 3 and 6 h after. Before any assessment, subjects were acclimatized for 20 min under controlled conditions (temperature 22°C ± 2°; hygrometry 45% ± 10%).

#### 2.3.2 Mechanical Aggression by Abrasion (Study 3)

In a single‐blind, non‐invasive, intra‐individual, randomized study (study 3; Table 1), a neutral cleanser was used throughout the study. Thirty European women who met the inclusion criteria were recruited, aged between 55 and 65 years old, and one outlier was excluded from the analysis (Study 3; Table 1). After a 7‐day wash‐out period, one designated zone per forearm was randomized to undergo abrasion performed by a trained technician applying constant pressure and 8 back‐and‐forth movements of emery paper (grain size 120, attached to a hard support).

The serum formula was applied on one randomized forearm twice daily (morning and evening) for 28 days, and the other forearm was the untreated control. Emery paper abrasion was performed on D0 and D28, and TEWL measurements were done before abrasion and 3 min after abrasion.

#### 2.3.3 Aggression by Tape Stripping Followed by Intense Cold or Hot Conditions (Studies 4 and 5)

Two single‐blind non‐invasive, monocentric, randomized, single‐blind studies were conducted in October 2022 in France (Studies 4 and 5; Table 1). In both studies, 40 European women who met inclusion criteria, aged between 18 and 65 years old were recruited (Studies 4 and 5; Table 1).

After 30 min stabilization in a controlled‐conditions measuring room (20°C–23°C, 40%–60% hygrometry), TEWL was measured at baseline before stripping (T0 pre‐stripping). Forearm skin was tape stripped (Monaderm, Monaco) with a mean number of tape strips per zone of 18–19 for Studies 4 and 18 for Study 5. TEWL was measured (T0 post‐stripping) before serum application and before exposure to intense temperatures. A technician applied 2 mg/cm^2^ of serum with a single‐use fingerstall on the designated zone on one forearm after tape stripping but before being placed in the controlled climate room. The other forearm of the volunteer remained untreated.

In Study 4 evaluating the effect of serum in cold, dry, and windy intense conditions after tape stripping, subjects were placed in a controlled room at +4°C ± 1°C, 0.88 m/s wind, low hygrometry.

In Study 5 evaluating the effect of serum in hot, dry, and windy intense conditions after tape stripping, subjects were placed in a controlled room at 40°C, 15% hygrometry for 1 and 2 h.

In both studies, TEWL was measured after tape stripping followed by 1 h in intense cold or hot conditions (T1h post‐stripping) and then a 2 h in intense environmental conditions (T2h post‐stripping). All TEWL measurements were taken in the measuring room after acclimatization.

$$\begin{array}{c} \text{Mean}\;\%\;\text{change from baseline post}-\text{tape strip}\\ =\left(\mathrm{Tn}-\text{T0 post}-\text{stripping}\right)/\left(\text{T0 post}-\text{stripping}\right)\times 100 \end{array}$$

$$\begin{array}{c} \%\text{Recovery}=\left(\left(\mathrm{Tn}-\text{T0 post stripping}\right)/\right.\\ \left.\left(\text{T0 post}-\text{stripping}-\text{T0}\;\mathrm{pre}-\text{stripping}\right)\right)\times 100 \end{array}$$

### 2.4 Statistical Analysis

Sample size was calculated from the experience of previous studies. Descriptive statistics were provided for each parameter (effective, mean, mean difference, standard deviation (SD), minimum and maximum). The normality of differences was checked with the Shapiro–Wilk test (significance level *p* < 0.01). The significance threshold was set at 5% bilateral. The Student's *t*‐test or non‐parametric Wilcoxon test was performed for paired data.

### 2.5 Ethical Considerations

The authors confirm that the ethical policies of the journal, as noted on the journal's author guidelines page, have been adhered to. No ethical approval was required for these studies on a marketed cosmetic as they are considered non‐interventional studies. Each study was conducted in accordance with the principles expressed in the World Medical Association Declaration of Helsinki, local laws and regulations governing clinical studies, and Good Clinical Practice. All volunteers provided written informed consent prior to any study‐related procedure.

## 3 Results

### 3.1 Chemical Aggression by Harsh Cleanser in Women (Study 1 in Europe and Study 2 in Singapore)

#### 3.1.1 Demographic Characteristics

A total of 31 women were included of mean (SD) age 61.0 (± 3.1) years and Fitzpatrick skin type II (22 subjects [71%]) or III (9 subjects [29%]) living in Europe (Study 1).

A total of 40 women of mean (SD) age 59.35(± 2.91) years and phototype III (27 subjects [67.5%]) or II (13 subjects [32.5%]) were included in Singapore (Study 2).

#### 3.1.2 Efficacy

**FIGURE 1:**
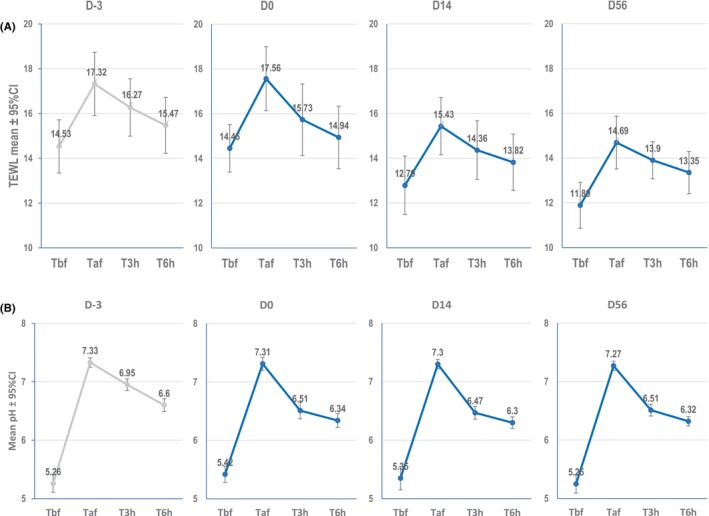
TEWL values (A) and pH values (B) before aggression (Tbf), immediately after chemical aggression by harsh cleanser (Taf) and then 3 (T3h) and 6 h (T6h) after aggression between D‐3 (untreated control) and D56 after 56 days of serum application, *n* = 31 women living in Europe (Study 1).

For both panels, after 14 days of application of the investigational serum formula, a significant decrease in the mean TEWL before cleanser aggression (Tbf) was observed compared to the untreated control at D‐3 (*p* = 0.00) and this effect was maintained at D56 (*p* < 0.0001 compared to D‐3) (Figure 1A example for Study 1 in Europe), indicating an improvement in skin barrier function due to the application of the serum formula.

The mean TEWL values increased immediately after aggression by the harsh cleanser (Taf) for all time points, but the changes after harsh cleanser aggression were significantly lower after 14 and 56 days of serum application compared to the untreated control at D‐3 (*p* = 0.001 and *p* < 0.001, respectively) (Figure 1A example for Study 1 in Europe), indicating a resistance against aggression.

After 14 and 56 days of serum application, TEWL was significantly lower at 3 h after harsh cleanser aggression compared to the untreated control (*p* < 0.0001 for 14 and 56 days, compared to D‐3) indicating a faster recovery after aggression.

The optimal acid pH for the skin at D‐3 before aggression (Tbf) was maintained throughout the study period from D0 to D56 with serum formula application (Figure 1B). Also, the serum formula had no significant effect on reducing the magnitude of skin pH alteration after aggression (Taf) compared to the control at D‐3 (Figure 1B example for Study 1 in Europe).

At 3 and 6 h after aggression by harsh cleanser, the skin pH was significantly lower at D0, D14, and D56 (for the European panel) and only after 56 days of application for the Singapore panel, compared to the untreated control at D‐3 (all *p* < 0.001 compared to baseline at D‐3) showing that serum application resulted in significantly better recovery of skin pH after harsh cleanser aggression (Figure 1B example for study 1 in Europe).

After application of the serum, a significantly faster recovery of the skin pH after harsh cleanser aggression was shown by slopes 1.3 times steeper compared to untreated skin at D‐3 (all *p* < 0.05 compared to D‐3) after one application for the European panel and by slopes 1.7 times steeper after 56 days of application for the Singapore panel.

### 3.2 Mechanical Aggression by Abrasion (Study 3)

#### 3.2.1 Demographic Characteristics

The mean (SD) age was 41 (± 11) years. One subject was excluded from the analysis due to outlier TEWL values.

#### 3.2.2 Efficacy

The standardized abrasion method increased TEWL at D0 and D28 on both the serum‐treated and untreated sides. At D0, the increase in TEWL was not statistically different between the treated and the control sides.

**FIGURE 2:**
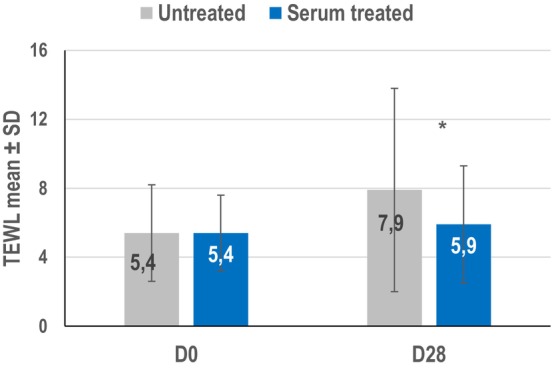
Difference in TEWL measurements before and after emery paper abrasion at baseline and after 28 days of serum application (*n* = 29) (Study 3). *p < 0.05.

After 28 days of application of the serum, the TEWL increase caused by abrasion was significantly lower, showing the skin was significantly less reactive to abrasion compared to the untreated control skin (*p* < 0.0475) (Table 2 and Figure 2). The difference in post‐abrasion TEWL increase between D0 and D28 was significantly greater at 2.5 ± 1.0 for the untreated side than for the serum‐treated side at 0.5 ± 0.8 (*p* = 0.0435) indicating that after 28 days of application of serum, the skin was more stable and more resistant to aggression.

**TABLE 2:** TEWL (g/m^2^/h) before and after mechanical abrasion on forearm skin and with or without 28 days of serum treatment (Study 3; *n* = 29).

Mean ± SD [95% CI]	Untreated control	Serum treated	*p* value
Day 0, *n* = 29			
Before abrasion	8.0 ± 1.5 [7.4, 8.5]	8.1 ± 1.7 [7.5, 8.7]	
After abrasion with emery paper	13.3 ± 3.3 [12.1, 14.6]	13.5 ± 2.8 [12.5, 14.6]	
Increase due to abrasion	**5.4 ± 2.8 [4.3, 6.4]**	**5.4 ± 2.2 [4.6, 6.3]**	NS
Day 28, *n* = 29			
Before abrasion	8.0 ± 1.3 [7.5, 8.5]	7.7 ± 1.5 [7.1, 8.3]	
After abrasion with emery paper	15.9 ± 6.3 [13.5, 18.3]	13.6 ± 3.9 [12.1, 15.1]	
Increase due to abrasion	7.9 ± 5.9 [5.7, 10.2]	5.9 ± 3.4 [4.6, 7.2]	0.0475
Difference in increase due to abrasion D28‐D0	2.5 ± 1.0	0.5 ± 0.8	0.0435

*Note:* Bold values results non significant (NS).

### 3.3 Aggression by Tape Stripping Followed by Intense Cold or Hot Conditions (Studies 4 and 5)

#### 3.3.1 Demographic Characteristics

The mean (SD) age was 42 (± 12) years for the study in intense cold conditions and 41 (± 10) years for the study in intense hot conditions.

#### 3.3.2 Efficacy in Intense Cold Conditions

**FIGURE 3:**
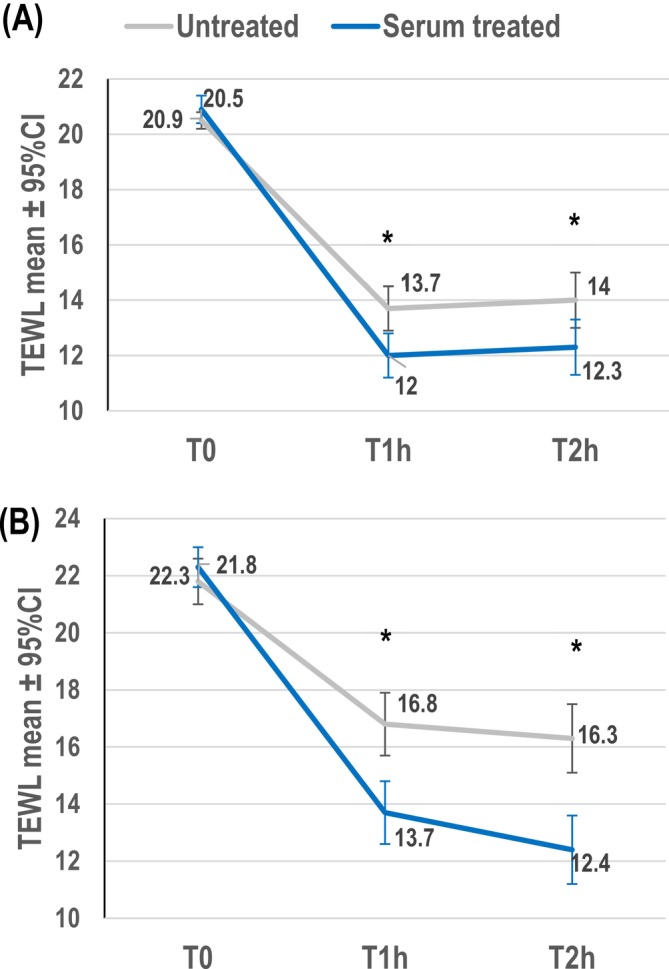
Mean TEWL measurements after aggression by tape stripping (T0) followed by 1 h (T1h) to 2 h (T2h) in intense cold at 4°C and windy conditions (*n* = 24) (A; Study 4) or intense heat at 40°C, dry and windy conditions (*n* = 24) (B; Study 5) for the serum treated and untreated sites. *Significant difference between serum treated and control site (Student test *p* < 0.0001).

After tape stripping and 1 or 2 h exposure to intense cold conditions, the TEWL values were statistically significantly lower for the zone treated with a single application of serum formula than the untreated zone (*p* < 0.0001 for both 1 and 2 h timepoints) (Table 3 and Figure 3). The serum formula improved skin barrier function recovery, showing a better repair efficacy in barrier function in intense cold conditions, compared to the untreated control (Table 3). The variation in mean TEWL between baseline and 1 h after application of the serum was 9.6% lower in the treated zone compared to the untreated control zone (Wilcoxon test, *p* value < 0.0001). The variation in mean TEWL between baseline and 2 h after application of the serum was 9.2% lower in the treated zone compared to the untreated control zone (Student test, *p* value < 0.0001).

**TABLE 3:** TEWL (g/m^2^/h) results after tape stripping aggression followed by intense cold (4°C), windy conditions (Study 4) or hot (40°C) dry and windy conditions (Study 5).

Mean ± SD [95% CI]	Untreated control	Serum treated	*p* value	Variation in mean TEWL between baseline and after cold conditions, between treated and untreated zone
Cold conditions, *n* = 24				
T0 before tape stripping	10.1 ± 2.0 [9.3, 11.0]	10.2 ± 2.0 [9.3, 11.0]	NS	
T0 after tape stripping	20.5 ± 0.8 [20.2, 20.9]	20.9 ± 1.1 [20.4, 21.3]	NS	
1 h in cold conditions	13.7 ± 1.9 [12.9, 14.6]	12.0 ± 1.7 [11.2, 12.7]		
Mean change from baseline after tape stripping and 1 h in cold conditions	−6.8 ± 2.1 [−7.7, −5.9]	−8.9 ± 1.7 [−9.6, −8.2]	**< 0.0001**	
Mean % change from baseline after tape stripping and 1 h in cold conditions	−33,1%	−42.1%	**< 0.0001**	−**9.6%**
% recovery from baseline after tape stripping and 1 h in cold conditions	−65.1%	−83.3%		
2 h in cold conditions	14.0 ± 2.4 [13.0, 15.0]	12.3 ± 2.5 [11.3, 13.4]		
Mean change from baseline after tape stripping and 2 h in cold conditions	−6.5 ± 2.4 [−7.5, −5.5]	−8.6 ± 2.3 [−9.5, −7.6]	**< 0.0001**	
Mean % change from baseline after tape stripping and 2 h in cold conditions	−41%	−31,8%	**< 0.0001**	−**9.2%**
% recovery	−62.5%	−80%		

Mean ± SD [95% CI]	Untreated control	Serum treated	*p* value	Variation in mean TEWL between baseline and after cold conditions, between treated and untreated zone
Hot conditions, *n* = 24				
T0 before tape stripping	9.1 ± 1.8 [8.3; 9.9]	9.0 ± 1.9 [8.2; 9.8]	NS	
T0 after tape stripping	21.8 ± 1.8 [21.0; 22.5]	22.3 ± 1.6 [21.6; 22.9]	NS	
1 h in hot conditions	16.8 ± 2.7 [15.7; 18.0]	13.7 ± 2.5 [12.6; 14.8]		
Mean change from baseline after tape stripping and 1 h in hot conditions	−5.0 ± 2.7 [−6.1; −3.8]	−8.6 ± 3.4 [−10.0; −7.1]	**< 0.0001**	
Mean % change from baseline after tape stripping and 1 h in hot conditions	−22.8%	−38.5%	**< 0.0001**	**−15.7%**
% recovery from baseline after tape stripping and 1 h in hot conditions	−39.1%	−64.7%		
2 h in hot conditions	16.3 ± 2.7 [15.1; 17.4]	12.4 ± 2.9 [11.2; 13.6]		
Mean change from baseline after tape stripping and 2 h in hot conditions	−5.5 ± 3.0 [−6.8; −4.2]	−9.9 ± 3.8 [−11.5; −8.3]	< 0.0001	
Mean % change from baseline after tape stripping and 2 h in hot conditions	−25.3%	−44.4%	**< 0.0001**	**−19.1%**
% recovery from baseline after tape stripping and 2 h in hot conditions	−43.4%	−74.6%		

*Note:* Bold values are statistically significant.

#### 3.3.3 Efficacy in Intense Hot Conditions

After tape stripping and 1 or 2 h exposure to hot conditions, the TEWL values were statistically significantly lower in the zone treated with a single application of serum formula compared to the untreated zone (Table 3 and Figure 3). Compared to untreated skin, the serum formula improved skin quality with a higher percentage recovery, showing a better repair efficacy in barrier function in intense hot conditions (Table 3). The variation in mean TEWL between baseline and 1 h after application of the serum was 15.7% lower in the treated zone compared to the control (Wilcoxon test, *p* value < 0.0001). The variation in mean TEWL between baseline and 2 h after application of the serum was 19.1% lower in the treated zone compared to the control (Student test, *p* value < 0.0001).

## 4 Discussion

Although our studies have several limitations, the results of five clinical studies taken together showed that the tested serum formula, containing short and long saccharides and bacterial fractions, helped repair and strengthen the skin barrier after chemical aggression from a harsh cleanser, mechanical aggression from emery paper abrasion, and cumulative aggression from tape stripping followed by intense cold or hot environmental conditions.

First, in both harsh cleanser studies, including women from Europe of mainly Fitzpatrick skin type II and women from Singapore of mainly Fitzpatrick skin type III, serum application improved the baseline skin barrier function, as shown by lower TEWL before aggression on D56 compared to D0. Furthermore, although the serum application did not mitigate skin barrier dysfunction immediately after harsh cleanser aggression compared to the untreated control skin, skin barrier recovery was faster after 2 months of serum applications, as measured by TEWL and pH. Second, skin was less reactive after application of serum for 28 days, suggesting it had become more stable and more resistant to emery paper abrasion compared to untreated skin. Third, compared to untreated skin, a single application of the serum formula resulted in a significantly faster skin barrier recovery after cumulative aggression from tape stripping then intense cold or hot environmental conditions.

We studied three different aggressions that each challenge the skin's surface physiology, especially the skin barrier, in different ways. Indeed, harsh cleanser may affect the skin barrier function by damaging stratum corneum proteins, denaturing enzymes and altering the water‐holding capability of corneocytes [10]. Aggression by emery paper abrasion of the stratum corneum damages the underlying epidermal layers and potentially induces inflammation [11]. Conversely, tape stripping removes layers of the stratum corneum and may eventually remove lipids, ultimately disrupting their organization and compromising the skin's ability to retain moisture [12]. Furthermore, in our study, tape stripping was followed by intense cold or hot environmental conditions. Previous studies found that exposure to increased temperatures increased skin barrier dysfunction [13], while low temperatures lead to a decrease in skin hydration and TEWL [14]. A significant impact of seasonal change on skin barrier function has previously been reported [14, 15], highlighting the importance of testing formulations under various different environmental conditions.

When skin is exposed to internal or external aggressions, the skin barrier function and barrier repair may become impaired due to the formation of a larger number of smaller immature corneocytes, especially in individuals with sensitive skin [16, 17]. For example, mechanical assaults by physical aggressors (e.g., harsh cleanser, abrasion) and extreme environmental conditions (e.g., pollution, sunlight, seasonal changes, hot, cold, windy, humid environmental conditions) can damage the skin barrier. Similarly, psychological stressors have an impact on epidermal barrier function by activating the hypothalamic–pituitary–adrenal axis to stimulate local and systemic production of stress hormones. Stress hormones adversely affect the epidermal barrier by decreasing epidermal lipids and structural proteins, decreasing stratum corneum hydration, and increasing TEWL [18].

Physical or mechanical aggression can disrupt the skin barrier, especially if it is already weakened by other types of aggression from cumulative exposome factors. In particular, skin function is affected by urban pollution or lifestyle factors over an individual's lifetime [5]. Air pollutants damage the skin by inducing oxidative stress, highlighting the importance of skin care containing bacterial fractions with antioxidant capacity [19].

Skin aging is a complex biological process influenced by a combination of endogenous and exogenous factors [18], and different aggressions may contribute to skin aging by several mechanisms [20]. Over the long term, these factors lead to structural and physiological alterations in each skin layer and visible skin aging. Using skin care to mitigate skin barrier dysfunction from aggressions can improve skin health and quality and thus help prevent the appearance of skin aging signs.

Previous data in 30 healthy volunteers showed that application for 15 days of a prebiotic skin care formula containing *Lactobacillus* extracts and long and short saccharides helped restore microbial diversity and induced improvement in skin quality and resistance to aggressions from soap cleansing [21]. Bacterial fractions are known to decrease TEWL, as we observed with the serum formula, improve skin dryness, and strengthen the skin barrier function [22, 23]. It has also been suggested that bacterial fractions may help restore disturbed skin microbiota following aggression and thereby influence epithelial and immune cell function [24]. Furthermore, the interplay between the skin barrier and the skin (and gut) microbiota may influence the skin aging process [25]. As physiological changes in the skin with aging may affect the skin microbiome and its function, the development of new microbiome‐based solutions may improve skin quality and appearance [26].

## 5 Study Limitations

The main limitation of these intra‐individual studies is the lack of a control group with a vehicle serum. As we only evaluated the complete serum formulation (with untreated skin as a control), we cannot draw conclusions on the role of ingredients alone. However, 177 subjects were included in total across all studies, and the intra‐individual comparison of serum‐treated versus untreated skin consistently showed good effectiveness of the serum to protect and repair the skin barrier before and after exposure to aggression. Furthermore, we have previously shown the effectiveness of *Bifidobacterium longum* lysate on several barrier function markers in both in vitro and in vivo studies [8, 9]. Future studies are now warranted to compare the test serum formula, containing a unique combination of seven short and long saccharides and bacterial fractions, versus a control serum formula without active ingredients.

## 6 Conclusions

Internal and external aggressors (e.g., air pollution, intense temperature), whether acute or chronic exposure, are known to damage the skin barrier [5, 6].

The investigational serum formula, containing a unique combination of seven short and long saccharides and bacterial fractions, consistently showed efficacy in repairing and strengthening the skin barrier after exposure to different aggressions by harsh cleanser, abrasion, or tape stripping followed by intense cold or hot conditions, and is thus essential to maintaining skin health and quality.

## Author Contributions

**Caroline Pichon:** study design, writing – review and editing. **Marion Mesrobian:** study design, study management, writing – review and editing. **Sybille De Bussy:** study design, study management, writing – review and editing. **Elodie Valverde:** review and editing. **Audrey Gueniche:** study design, study management, writing – review and editing.

## Ethics Statement

The authors confirm that the ethical policies of the journal, as noted on the journal's author guidelines page, have been adhered to. No ethical approval was required for these studies on a marketed cosmetic as they are considered non‐interventional studies. Each study was conducted in accordance with the principles expressed in the World Medical Association Declaration of Helsinki, local laws and regulations governing clinical studies, and Good Clinical Practice. All volunteers provided written informed consent prior to any study‐related procedure.

## Conflicts of Interest

All authors are employees of L'Oréal Research and Innovation.

## Data Availability

The data that support the findings of this study are available from the corresponding author upon reasonable request.

## References

[1] J. A. Bouwstra , F. E. Dubbelaar , G. S. Gooris , A. M. Weerheim , and M. Ponec , “The Role of Ceramide Composition in the Lipid Organisation of the Skin Barrier,” Biochimica et Biophysica Acta 1419, no. 2 (1999): 127–136, 10.1016/s0005-2736(99)00057-7. 10407065

[2] S. Verdier‐Sévrain and F. Bonté , “Skin Hydration: A Review on Its Molecular Mechanisms,” Journal of Cosmetic Dermatology 6, no. 2 (2007): 75–82, 10.1111/j.1473-2165.2007.00300.x. 17524122

[3] J. W. Fluhr , K. R. Feingold , and P. M. Elias , “Transepidermal Water Loss Reflects Permeability Barrier Status: Validation in Human and Rodent In Vivo and Ex Vivo Models,” Experimental Dermatology 15, no. 7 (2006): 483–492, 10.1111/j.1600-0625.2006.00437.x. 16761956

[4] A. L. Byrd , Y. Belkaid , and J. A. Segre , “The Human Skin Microbiome,” Nature Reviews. Microbiology 16, no. 3 (2018): 143–155, 10.1038/nrmicro.2017.157. 29332945

[5] T. Passeron , J. Krutmann , M. L. Andersen , R. Katta , and C. C. Zouboulis , “Clinical and Biological Impact of the Exposome on the Skin,” Journal of the European Academy of Dermatology and Venereology 34, no. Suppl 4 (2020): 4–25, 10.1111/jdv.16614. 32677068

[6] T. Passeron , C. C. Zouboulis , J. Tan , et al., “Adult Skin Acute Stress Responses to Short‐Term Environmental and Internal Aggression From Exposome Factors,” Journal of the European Academy of Dermatology and Venereology 35, no. 10 (2021): 1963–1975, 10.1111/jdv.17432. 34077579 PMC8519049

[7] J. Dou , N. Feng , F. Guo , et al., “Applications of Probiotic Constituents in Cosmetics,” Molecules 28, no. 19 (2023): 6765, 10.3390/molecules28196765. 37836607 PMC10574390

[8] A. G. Szöllősi , A. Gueniche , O. Jammayrac , et al., “ *Bifidobacterium longum* Extract Exerts Pro‐Differentiating Effects on Human Epidermal Keratinocytes, In Vitro,” Experimental Dermatology 26, no. 1 (2017): 92–94, 10.1111/exd.13130. 27315170

[9] A. Guéniche , P. Bastien , J. M. Ovigne , et al., “ *Bifidobacterium longum* Lysate, a New Ingredient for Reactive Skin,” Experimental Dermatology 19, no. 8 (2010): e1–e8, 10.1111/j.1600-0625.2009.00932.x. 19624730

[10] Z. D. Draelos , “The Science Behind Skin Care: Cleansers,” Journal of Cosmetic Dermatology 17, no. 1 (2018): 8–14, 10.1111/jocd.12469. 29231284

[11] C. Y. Hsieh and T. F. Tsai , “Friction‐Aggravated Skin Disorders—A Review of Mechanism and Related Diseases,” Dermatitis 34, no. 4 (2023): 287–296, 10.1097/der.0000000000000961. 36255396

[12] J. W. Fluhr , H. Dickel , O. Kuss , I. Weyher , T. L. Diepgen , and E. Berardesca , “Impact of Anatomical Location on Barrier Recovery, Surface pH and Stratum Corneum Hydration After Acute Barrier Disruption,” British Journal of Dermatology 146, no. 5 (2002): 770–776, 10.1046/j.1365-2133.2002.04695.x. 12000372

[13] J. W. Hui‐Beckman , E. Goleva , D. Y. M. Leung , and B. E. Kim , “The Impact of Temperature on the Skin Barrier and Atopic Dermatitis,” Annals of Allergy, Asthma & Immunology 131, no. 6 (2023): 713–719, 10.1016/j.anai.2023.08.007. 37595740

[14] K. A. Engebretsen , J. D. Johansen , S. Kezic , A. Linneberg , and J. P. Thyssen , “The Effect of Environmental Humidity and Temperature on Skin Barrier Function and Dermatitis,” Journal of the European Academy of Dermatology and Venereology 30, no. 2 (2016): 223–249, 10.1111/jdv.13301. 26449379

[15] J. A. Segre , “Epidermal Barrier Formation and Recovery in Skin Disorders,” Journal of Clinical Investigation 116, no. 5 (2006): 1150–1158, 10.1172/jci28521. 16670755 PMC1451215

[16] N. Raj , R. Voegeli , A. V. Rawlings , et al., “A Fundamental Investigation Into Aspects of the Physiology and Biochemistry of the Stratum Corneum in Subjects With Sensitive Skin,” International Journal of Cosmetic Science 39, no. 1 (2017): 2–10, 10.1111/ics.12334. 27079667

[17] A. Pons‐Guiraud , “Sensitive Skin: A Complex and Multifactorial Syndrome,” Journal of Cosmetic Dermatology 3, no. 3 (2004): 145–148, 10.1111/j.1473-2130.2004.00082.x. 17134429

[18] M. Maarouf , C. L. Maarouf , G. Yosipovitch , and V. Y. Shi , “The Impact of Stress on Epidermal Barrier Function: An Evidence‐Based Review,” British Journal of Dermatology 181, no. 6 (2019): 1129–1137, 10.1111/bjd.17605. 30614527

[19] E. Araviiskaia , E. Berardesca , T. Bieber , et al., “The Impact of Airborne Pollution on Skin,” Journal of the European Academy of Dermatology and Venereology 33, no. 8 (2019): 1496–1505, 10.1111/jdv.15583. 30897234 PMC6766865

[20] K. E. Burke , “Mechanisms of Aging and Development‐A New Understanding of Environmental Damage to the Skin and Prevention With Topical Antioxidants,” Mechanisms of Ageing and Development 172 (2018): 123–130, 10.1016/j.mad.2017.12.003. 29287765

[21] A. Gueniche , C. Clavaud , O. Perin , et al., “A Skin Prebiotic Cream for Helping the Microbiome Recovery After Soap Cleansing”.

[22] T. Gao , X. Wang , Y. Li , and F. Ren , “The Role of Probiotics in Skin Health and Related Gut‐Skin Axis: A Review,” Nutrients 15, no. 14 (2023): 3123, 10.3390/nu15143123. 37513540 PMC10385652

[23] C. R. Harding , A. Watkinson , A. V. Rawlings , and I. R. Scott , “Dry Skin, Moisturization and Corneodesmolysis,” International Journal of Cosmetic Science 22, no. 1 (2000): 21–52, 10.1046/j.1467-2494.2000.00001.x. 18503460

[24] G. Wieërs , L. Belkhir , R. Enaud , et al., “How Probiotics Affect the Microbiota,” Frontiers in Cellular and Infection Microbiology 9 (2019): 454, 10.3389/fcimb.2019.00454. 32010640 PMC6974441

[25] Y. R. Woo and H. S. Kim , “Interaction Between the Microbiota and the Skin Barrier in Aging Skin: A Comprehensive Review,” Frontiers in Physiology 15 (2024): 1322205, 10.3389/fphys.2024.1322205. 38312314 PMC10834687

[26] T. Myers , A. Bouslimani , S. Huang , et al., “A Multi‐Study Analysis Enables Identification of Potential Microbial Features Associated With Skin Aging Signs,” Frontiers in Aging 4 (2023): 1304705, 10.3389/fragi.2023.1304705. 38362046 PMC10868648

